# Supplementary material for: The relationship between digital media use during pregnancy, maternal psychological wellbeing, and maternal-fetal attachment
Source: PLoS One. 2020 Dec 16;15(12):e0243898. doi: 10.1371/journal.pone.0243898 (PMC7743947; doi:10.1371/journal.pone.0243898)
Supplement: S1 Appendix — (DOCX) [file pone.0243898.s001.docx]

**S1 Appendix. Digital media questionnaire**

1. What forms of digital media do you use during this pregnancy? Please either select “Do not use” or rank your usage from 1 – 8 (1 = being most frequently used, 8 = being least frequently used)

| - Facebook | - YouTube |
| --- | --- |
| - Facebook Messenger | - Instagram |
| - Parenting websites | - Online parenting forums |
| - Smart phone applications (i.e. Apps) - Search Engines, eg. Google. | - Other, please specify… |
|  |  |

1. Please rate in order of 1^st^ (Most important) to 6^th^ (Least important) in terms of where you would go for pregnancy advice and resources?

- Social media
- Parenting blogs
- General practitioner
- Medical specialist
- Family/friend
- Books

1. What are your main reasons for using digital media during this pregnancy? Please check as many as applicable.

| - Information seeking | - Understanding the development of my baby |
| --- | --- |
| - Social support | - To feel closer to my baby |
| - Emotional Support | - Connecting with family and friends |
| - Medical advice | - Reassurance |
| - Sharing photos | - To pass time |
| - Other, please specify… |  |

1. Do you use digital media to find information regarding the development of your fetus during this pregnancy? If yes, please provide examples of what you search for most often.
2. Do you use digital media to find information regarding your health during this pregnancy? If yes, please provide examples of what you search for most often.
3. When are you most likely to use digital media to find information during pregnancy?

- When experiencing a symptom for the first time
- Between doctor/midwife appointments
- Before or after an ultrasound
- When hearing conflicting advice from friends/family
- At random stages during pregnancy, i.e. no specific circumstances
- None of the above

1. Are you likely to use digital media for information seeking before a doctor/midwife appointment? If so, what are your reasons for doing this?

| - Yes | - No |
| --- | --- |
| - - To prepare questions |  |
| - - To ease nerves about not knowing what to expect   - To feel knowledgeable |  |
| - - Other, please specify… |  |

1. Are you likely to use digital media for information seeking after a doctor/midwife appointment? If so, what are your reasons for doing this?

| - Yes | - No |
| --- | --- |
| - - To clarify what was said |  |
| - - For reassurance   - For a second opinion |  |
| - - Other, please specify… |  |

1. How often do you use digital media for information seeking during this pregnancy? Please select one.

| - More than five (5) times a day | - Two (2) to five (5) times a day |
| --- | --- |
| - Once a day | - Three (3) to five (5) times a week |
| - One (1) to two (2) times a week | - Less than once a week |
|  |  |
|  |  |

1. How often do you use digital media for social or emotional support during this pregnancy? Please select one.

| - More than five (5) times a day | - Two (2) to five (5) times a day |
| --- | --- |
| - Once a day | - Three (3) to five (5) times a week |
| - One (1) to two (2) times a week | - Less than once a week |
|  |  |
|  |  |

1. How often would you share photos or updates of your pregnancy on your social networking site/s?

- Never
- Once a month
- Once a week
- Once a day
- More than once a day

1. In your opinion, do your friends’ social media profiles accurately portray their lives?

- Yes
- No
- I don’t think about this


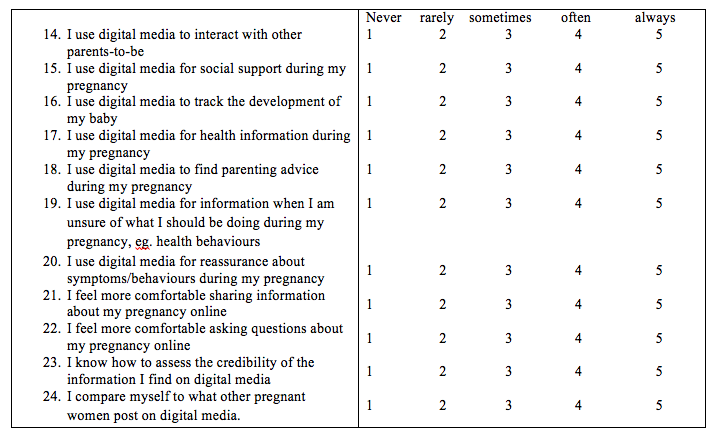


1. In your opinion, do your friends’ social media profiles accurately portray their pregnancies?

- Yes
- No
- I don’t think about this
