# Supplementary material for: The relationship between digital media use during pregnancy, maternal psychological wellbeing, and maternal-fetal attachment
Source: PLoS One. 2020 Dec 16;15(12):e0243898. doi: 10.1371/journal.pone.0243898 (PMC7743947; doi:10.1371/journal.pone.0243898)
Supplement: Attachment — Submitted filename: Response to Reviewers.docx [file pone.0243898.s003.docx]

**PONE-D-20-05764**

**The relationship between digital media use during pregnancy, maternal psychological wellbeing, and maternal-fetal attachment**

**Christine E East**

**Academic Editor**

**PLOS ONE**

Dear Christine E East,

We appreciate your feedback and opportunity to revise our manuscript for submission at PLOS ONE. We have responded to each comment below, with line number references given to relevant changes in the attached ‘Revised Manuscript with Tracked Changes’ document.

*General comments:*

- *Although PLOS One does not specify this, I would encourage you to avoid the term “statistically significant” and replace it with commentary like “there was limited evidence to support…” or “we found no evidence of a difference …” See https://community.cochrane.org/sites/default/files/uploads/inline-files/Interpreting%20statistical%20significance.pdf*
- *For example, in line 14 of the Abstract, you could write: “There was no evidence of a relationship between digital media use and maternal-fetal attachment” (and give data as evidence, see below)*
- *Referencing in text: The Vancouver style, as I understand it, only imports the reference number in sequence as you have mostly done, rather than listing more than one author. For example, line 45, “Akiki, Avison [13] found …” needs to read as “Akiki et al [13] found …”*

Authors’ response:

We have made your suggested changes to the language used when reporting statistical significance (examples of such changes on lines 307 – 310; 322). We have also updated the Vancouver referencing style in the manuscript to suit the journal’s referencing requirements.

*Abstract comments:*

- *The survey is noted, however, no detail is provided about how findings were analysed, eg, mean, SD, assigning “formulated meanings” to comments, etc. No actual results are provided, only a comment about no association.*
- *Measures, lines 131-134: I don’t understand how the questions used could result in free text responses. They appear to indicate the need for a “yes/no” response. How, then, did you ask women to provide free text?*

Authors’ response:

We have included further details about the key findings in the abstract, including means, standard deviations, and significance values. We have elaborated in the measures section of the methods, to clarify that the questionnaire had the option of providing free text qualitative response (see lines 147 – 150). The digital media use questionnaire is now included as supporting information.

*Results comments:*

- *Table 1 is usually the summary of participants’ characteristics. You could then reduce the commentary of these in text.*
- *The current Table 1 only contains a small amount of data, which could be combined with the data in Table 3. Indeed, much of the text could be converted to table here, also incorporating the linear relationship analyses – the latter are currently figures which would not add anything to a journal publication and need to be removed. The detail in Table 2 is interesting – however, it would be helpful to have more detail in the methods section about what “formulated meaning” is, how many people go through the comments to determine this and how consensus on these meanings is achieved. You could provide a second quote for each of the three areas here. Also, there could be the potential to provide some quotes that did not support the “formulated meanings” and how you dealt with these.*
- *In reporting mean and standard deviation, please use one decimal point for the mean and two for the SD. You can summarise the comments about not meeting normal distribution – you have noted that the test used was robust given the sample size. For the ANOVA results, you only need the p-value, rather than all the detail.*

Authors’ response:

Thank you for your recommendations to the results section. We have taken your feedback on adjusting mean and SD decimal points and re-considered how we presented information in-text versus table form. As there were several variables in participant characteristics that we believe would have been difficult to present in table form clearly we decided to leave Table 1 as is and the participant characteristics in written form,

We agree that the term formulated meanings was a poor choice. We have revised this section to explain how the thematic analysis was conducted in line with Braun and Clarke’s (2006) procedure. Further details are now provided in the methods data analysis section of the manuscript (see lines 220-226). We also provided additional quotes in Table 2 of the results to illustrate the themes and associated meanings we identified.

*Discussion comments:*

- *This is good overall.*
- *Line 320, the word “psychology” should be “psychological”.*
- *Line 381, the word “reflects” would be better as “aligns with” as you initiated this method, rather than it reflecting the mode of survey that participants responded to when provided with a choice of mode.*

Authors’ response:

We have revised these grammatical errors.

Kind regards,

Corresponding Author, Associate Professor Jane Herbert
